# Long-term survival among older patients with myocardial infarction differs by educational level: results from the MONICA/KORA myocardial infarction registry

**DOI:** 10.1186/1475-9276-13-19

**Published:** 2014-02-19

**Authors:** Inge Kirchberger, Christa Meisinger, Hildegard Golüke, Margit Heier, Bernhard Kuch, Annette Peters, Philip A Quinones, Wolfgang von Scheidt, Andreas Mielck

**Affiliations:** 1 Central Hospital of Augsburg, MONICA/KORA Myocardial Infarction Registry, Stenglinstr. 2, Augsburg D-86156, Germany; 2 Helmholtz Zentrum München, German Research Center for Environmental Health (GmbH), Institute of Epidemiology II, Ingolstädter Landstr. 1, Neuherberg 85764, Germany; 3 Department of Internal Medicine/Cardiology, Hospital of Nördlingen, Stoffelsberg 4, Nördlingen 86720, Germany; 4 Department of Internal Medicine I – Cardiology, Central Hospital of Augsburg, Stenglinstr. 2, Augsburg D-86156, Germany; 5 Helmholtz Zentrum München, German Research Center for Environmental Health, |Institute of Health Economics and Health Care Management, Ingolstädter Landstr. 1, Neuherberg 85764, Germany

**Keywords:** Socioeconomic status, Myocardial infarction, Mortality, Germany

## Abstract

**Background:**

Socioeconomic disparities in survival after acute myocardial infarction (AMI) have been found in many countries. However, population-based results from Germany are lacking so far. Thus, the objective of this study was to examine the association between educational status and long-term mortality in a population-based sample of people with AMI.

**Methods:**

The sample consisted of 2,575 men and 844 women, aged 28–74 years, hospitalized with a first-time AMI between 1 January 2000 and 31 December 2008, recruited from a population-based AMI registry. Patients were followed up until December 2011. Data on education, risk factors and co-morbidities were collected by individual interviews; data on clinical characteristics and AMI treatment by chart review. Cox proportional hazards models were used to assess the relationship between educational status and long-term mortality.

**Results:**

During follow-up, 19.1% of the patients with poor education died compared with 13.1% with higher education. After adjustment for covariates, no effect of education on mortality was found for the total sample and for patients aged below 65 years. In older people, however, low education level was significantly associated with increased mortality (hazard ratio (HR) 1.44, 95% confidence interval (CI) 1.05–1.98, p = 0.023). Stratified analyses showed that women older than 64 years with poor education were significantly more likely to die than women in the same age group with higher education (HR 1.57, 95% CI 1.02–2.41, p = 0.039).

**Conclusions:**

Elderly, poorly educated patients with AMI, and particularly women, have poorer long-term survival than their better educated peers. Further research is required to illuminate the reasons for this finding.

## Introduction

Cardiovascular diseases such as acute myocardial infarction (AMI) are a leading cause of death in industrialized countries [1]. Even though a general decline in cardiovascular mortality has been observed in the last three decades, several studies have indicated that this decline may not be evenly distributed in the population. Socioeconomically disadvantaged people are often reported to have shorter survival after AMI than other patients [2-17]. This increased mortality risk can be quite large; the highest rate ratio (RR = 11.13) has been reported in a recent study from Finland concerning 28-day deaths among women in the highest income sixth versus the lowest income sixth [18]. Not all studies confirm these findings. Some report no or weak associations between socioeconomic status (SES) and AMI mortality [19-24]. However, it is difficult to compare the results of these studies because they vary substantially in terms of country of origin, follow-up time and indicators used for assessing SES.

Available studies come from many different countries such as Scotland [25], Sweden [9,26-28], Denmark [6], Finland [16,18], The Netherlands [10,11], France [29], United Kingdom [21], Italy [3,8,17,19], Spain [24], Israel [5,30], the USA [4,12-14,23,30], Canada [15] and even from Iran [2]. No paper has yet been published from Germany, and it is important to stress that results from one country should not simply be applied to another country. Survival after AMI very much depends on health care provided to patients with AMI, and thus also reflects access to country-specific health care.

Most of the available studies on socioeconomic inequalities regarding survival after AMI considered only short-term case fatality, i.e. mortality after 1 day [25,26], after 28–30 days [6,8-13,17-19,23,25,27,28] or after 1 year [4,7,10,15,16,19,21,22],[31]. There are two studies with case fatality after 2 years [2,20] and another two with case fatality after 7 years [6,14]. Longer periods are only included in a study from Israel, covering a maximum of 13 years after AMI [5,30,32]. However, this study sample was restricted to those aged 65 years or less.

In a number of studies, SES is assessed on a regional level, for example by neighbourhood median income [15,31], regional deprivation [14,21,25,27] or statewide income inequality in the USA [23], leading to recommendations such as ‘health care should be improved in socially deprived regions’. In some studies, SES is assessed at the individual level, leading to recommendations such as ‘health care should be improved specifically for patients with low educational level’. Our study focuses on individual SES (i.e. educational level), and these studies show mixed results as well. For instance, Picciotto et al. [19] found no significant association between educational level and first-year mortality after adjustment for covariables, whereas Donyavi et al. [2] reported a 2.51-fold increased risk of dying within 3 years for illiterate patients compared with patients with higher education. The available studies also show that it is essential to adjust for relevant confounders, as almost all studies detected a significant crude association between SES and mortality, which often attenuates substantially after adjusting for covariables [12,22]. However, many studies have not comprehensively adjusted their results for cardiovascular risk factors, co-morbidities and clinical characteristics such as diabetes, smoking, left ventricular failure, revascularization therapy or recurrent AMI, all of which are important predictors of post-AMI survival and are reported to vary by SES [6,10,11,16,17].

The objective of the analyses presented below is to further clarify the association between individual SES and case fatality by adding results from a well-defined population-based study in Germany, based on a long-term mortality follow-up, controlling for a number of risk factors, co-morbidities, AMI characteristics and treatment variables.

### Patients and methods

The population-based Augsburg Myocardial Infarction Registry was established in 1984 as part of the WHO-MONICA (Monitoring Trends and Determinants in Cardiovascular Disease) project [33]. After the termination of MONICA in 1995, the registry became part of the KORA (Cooperative Health Research in the Region of Augsburg) framework. Since 1984, all cases of coronary deaths and non-fatal AMI in the 25- to 74-year-old study population in the city of Augsburg and the two adjacent counties (totalling 600,000 inhabitants) have been continuously registered. Patients hospitalized in eight hospitals within the study region and two hospitals in the adjacent areas are included. About 80% of all AMI cases in the study region are treated in the study region’s major hospital, Klinikum Augsburg, a tertiary care centre offering invasive and interventional cardiovascular procedures, as well as heart surgery facilities [33,34]. Methods of case identification, diagnostic classification of events and data quality control have been described elsewhere [33,34]. The study was approved by the ethics committee of the Bavarian chamber of physicians and performed in accordance with the Declaration of Helsinki. Participants gave written informed consent prior to study inclusion.

### Sample

This study includes all patients registered between 1 January 2000 and 31 December 2008, who survived longer than 28 days with an incident AMI. Data collected before 2000 were not considered, because the definition and treatment of AMI has changed substantially. The patients were followed up until December 2011. From 4,405 men and women with an incident AMI during the study period, we excluded all subjects who could not be interviewed (n = 911) or whose data on any of the covariables were incomplete (n = 75). Reasons for missing interviews were death (n = 5), patient declined an interview (n = 326), insufficient German language skills (n = 101), early discharge (n = 66), delayed case identification (n = 232) or poor health status such as impaired consciousness or orientation (n = 181). Subjects who were excluded from the study sample because no interview could be performed had a significantly increased risk of dying compared with the patients included in the sample (hazard ratio [HR] 2.62; 95% confidence interval [CI] 2.26–3.03; p < 0.001). Finally, the present analyses comprised 3,419 people aged 28–74 years with an incident AMI.

### Data collection

Study participants were interviewed during their hospital stay after transfer from the intensive care unit, using a standardized questionnaire. The interviews were performed by trained study nurses and covered demographic information, risk factors and co-morbidities. Information on AMI characteristics, treatment and in-hospital complications were determined by chart review.

Educational level was selected as an indicator of SES. In German studies on health inequalities, educational level is widely accepted as being the most important indicator of SES for two reasons: educational level is crucial for future occupation, and educational level rarely changes after 20 years of age. SES was assessed by combining information on school education and vocational training, both gathered from the patient interview, reflecting the standard levels in the German educational system. The main characteristics of the German educational system are: three levels of school (in German: ‘Haupt-/Volksschule’, ‘Mittlere Reife’, ‘Abitur’) with the highest level (i.e. ‘Abitur’, usually reached after 13 years in school) qualifying for university, and three levels of vocational training: no vocational training, blue collar, white collar (e.g. university degree). It is difficult to fit the German educational system into the International Standard Classification of Education (ISCED). Great efforts have been made, for example in a recent international study [35]; finally, ‘Haupt-/Volksschule’ has been categorized as ISCED 2 or 3, depending on the level of vocational training. In our study, ‘low education’ was defined as ‘Haupt-/Volksschule’ without completed formal vocational training, corresponding to ISCED 2.

For some statistical analyses, patients were divided into two age groups. The cut-off of ‘65 years’ has been chosen because retirement usually starts at this age in Germany and other studies have also used the same cut-off [6,12].

The following further potential confounders were collected: patients were asked whether they currently live alone (yes/no), they have ever smoked or have stopped smoking (current smoker/ex-smoker/never smoked) and whether they were diagnosed as having angina pectoris, high blood pressure, high blood lipids or blood glucose prior to the AMI event. Self-reported history of angina pectoris, hypertension, hyperlipidaemia or diabetes (yes/no) was only considered if the chart review confirmed these diseases. The history of stroke (yes/no) was only determined by self-report. Body mass index (BMI) was determined by assessment of height and weight during the hospital stay. Obesity (yes/no) was defined as BMI > 30 kg/m^2^. Application of any reperfusion therapy (yes/no) was defined as having received thrombolysis, percutaneous transluminal coronary angioplasty with or without stenting or coronary artery bypass surgery during the hospital stay. Information on AMI type (ST-segment elevation myocardial infarction, non-ST-segment elevation myocardial infarction, bundle branch block) was documented in the patients’ medical records. Reduced left ventricular ejection fraction (LVEF) was stated if echocardiography, ventriculography or radionuclide ventriculography performed during the hospital stay revealed a LVEF < 30% (yes/no). Several in-hospital complications were documented on the patients’ medical charts. As most of them were too infrequent to be analysed as single covariables and others, such as cardiac arrest, were intermediate variables to the outcome, a summary variable was built, indicating the presence (yes/no) of any of the following in-hospital complications: pulmonary oedema, cardiogenic shock, re-infarction, ventricular tachycardia and bradycardia.

The study end-point was all-cause mortality during a median follow-up period of 6.1 years. The observation time ranged from 33 days to 12 years. Mortality was ascertained by checking the vital status of all registered people in the MONICA/KORA MI registry through the population registries inside and outside the study area until 31 December 2011. This procedure ensured that the vital status of cohort members who had moved out of the study area could also be assessed, resulting in almost complete follow-up (just five missing cases).

### Data analysis

Continuous data were expressed as median values with interquartile ranges (IQR) or mean and standard deviation, and categorical variables as percentages. The primary independent variable ‘education’ was cross-tabulated with potential covariates (including risk factors, co-morbidities, clinical and treatment characteristics). The Chi^2^ test was used to test for differences in frequencies, and Student’s t-test or Wilcoxon test for independent samples was used to test for differences in continuous variables between low and high education. All potential covariates were subjected to bivariate log-rank tests against survival. Correlations among covariates were examined using Phi or Cramer V coefficients.

The association between low versus high education and long-term mortality was investigated using Cox proportional hazards models. The proportional hazards assumption was tested for each variable graphically. It was valid for all variables except ‘living alone’ and ‘AMI type’ used in the Cox models, shown by parallel lines of log (-log(event)) versus log of event times. Therefore, we performed additional analyses with these variables included as time-dependent covariables, but these new covariables were not significant and the mortality risk for low versus higher education was very similar to the results presented above.

Four Cox proportional hazards models were calculated for the total sample and for groups stratified by age and sex. First, the crude association between low versus high education and mortality was calculated. Second, the crude association was adjusted for age and sex. The third, ‘full model’ includes all covariables that were significantly (p < 0.2) associated with survival in the bivariate analysis in addition to age and sex. Finally, a parsimonious model was built by backward selection. This model only includes variables that significantly (p < 0.05) contribute to the model. Age and sex were forced to stay in the model. In order to control for potential cohort effects, we tested whether the year of AMI had an influence on the association between educational level and mortality, but no effects were found.

Interaction effects of age and sex and of education with all covariables mentioned above were tested, but failed to reach statistical significance (p < 0.05). In addition to analyses stratified by sex and age group, full Cox models were calculated separately for follow-up periods of 1 to 12 years, in 1-year steps.

## Results

The study sample consisted of 2,575 men and 844 women with a median age of 60 years. Further characteristics are detailed in Table 1. The univariate comparison between the two educational groups resulted in significant differences in a number of sociodemographic characteristics, risk factors and co-morbidities (see Table 1).

**Table 1:** Sample characteristics

	**Total sample**	**Low education**	**Higher education**	**p-value**
	**(n = 3,419)**	**(n = 490)**	**(n = 2,929)**	
**Sociodemographic characteristics**				
Female	844 (24.7)	313 (63.9)	531 (18.1)	<0.001
Age [years], mean ± SD	60.0 ± 9.7	63.8 ± 8.8	59.3 ± 9.7	<0.001
≤ 65 years	2,212 (64.7)	232 (47.4)	1,980 (67.6)	<0.001
> 65 years	1,207 (35.3)	258 (52.7)	949 (32.4)	
Living alone	614 (18.0)	116 (23.7)	498 (17.0)	<0.001
**Risk factors and co-morbidities**				
Diabetes	958 (28.0)	181 (36.9)	777 (26.5)	<0.001
Angina pectoris	491 (14.4)	86 (17.6)	405 (13.8)	0.030
Hypertension	2,601 (76.1)	411 (83.9)	2,190 (74.8)	<0.001
Hyperlipidaemia	2,445 (71.5)	355 (72.5)	2,090 (71.4)	0.620
Body mass index > 30 kg/m^2^	849 (24.8)	164 (33.5)	685 (23.4)	<0.001
Stroke	181 (5.3)	38 (7.8)	143 (4.9)	0.009
Smoking				
Current smoker	1,245 (36.9)	151 (31.2)	1,094 (37.8)	<0.001
Ex-smoker	1,048 (31.0)	102 (21.1)	946 (32.7)	
Never smoker	1,085 (32.1)	231 (47.7)	854 (29.5)	
**AMI characteristics and treatment**				
ST-segment elevation MI	1,372 (40.1)	203 (41.4)	1,169 (39.9)	0.688
Non-ST-segment elevation MI	1,879 (55.0)	266 (54.3)	1,613 (55.1)	
Bundle branch block	168 (4.9)	21 (4.3)	147 (5.0)	
Prehospital delay time [minutes], median/IQR	170/516	180/436	168.5/539	0.783
Any reperfusion treatment	2,934 (85.8)	408 (83.3)	2,526 (86.2)	0.081
Coronary artery bypass grafting	529 (15.5)	69 (14.1)	460 (15.7)	0.358
PTCA^a^ without stenting	166 (4.9)	28 (5.7)	138 (4.7)	0.335
PTCA with stenting	2,199 (64.4)	304 (62.1)	1,895 (64.7)	0.275
LVEF^b^ < 30%	251 (10.5)	27 (8.0)	224 (10.9)	0.105
**In-hospital complications**				
Any in-hospital complication^c^	391 (11.4)	51 (10.4)	340 (11.6)	0.440
Cardiac arrest	131 (3.8)	23 (4.7)	108 (3.7)	0.271
Pulmonary oedema	70 (2.1)	11 (2.3)	59 (2.0)	0.734
Bradycardia (< 50/min)	219 (6.4)	27 (5.5)	192 (6.6)	0.381
Re-infarction	71 (2.1)	11 (2.3)	60 (2.1)	0.774
Ventricular tachycardia	59 (1.7)	9 (1.8)	50 (1.7)	0.835
Ventricular fibrillation	89 (2.6)	15 (3.1)	74 (2.5)	0.491
Cardiogenic shock	70 (2.1)	8 (1.6)	62 (2.1)	0.487

^a^Percutaneous transluminal coronary angioplasty.

^b^Left ventricular ejection fraction.

^c^Includes pulmonary oedema, re-infarction, bradycardia, ventricular tachycardia and cardiogenic shock.

Number and percentage of patients (p-values refer to tests of differences between patients with low versus high education).

**Figure 1:**
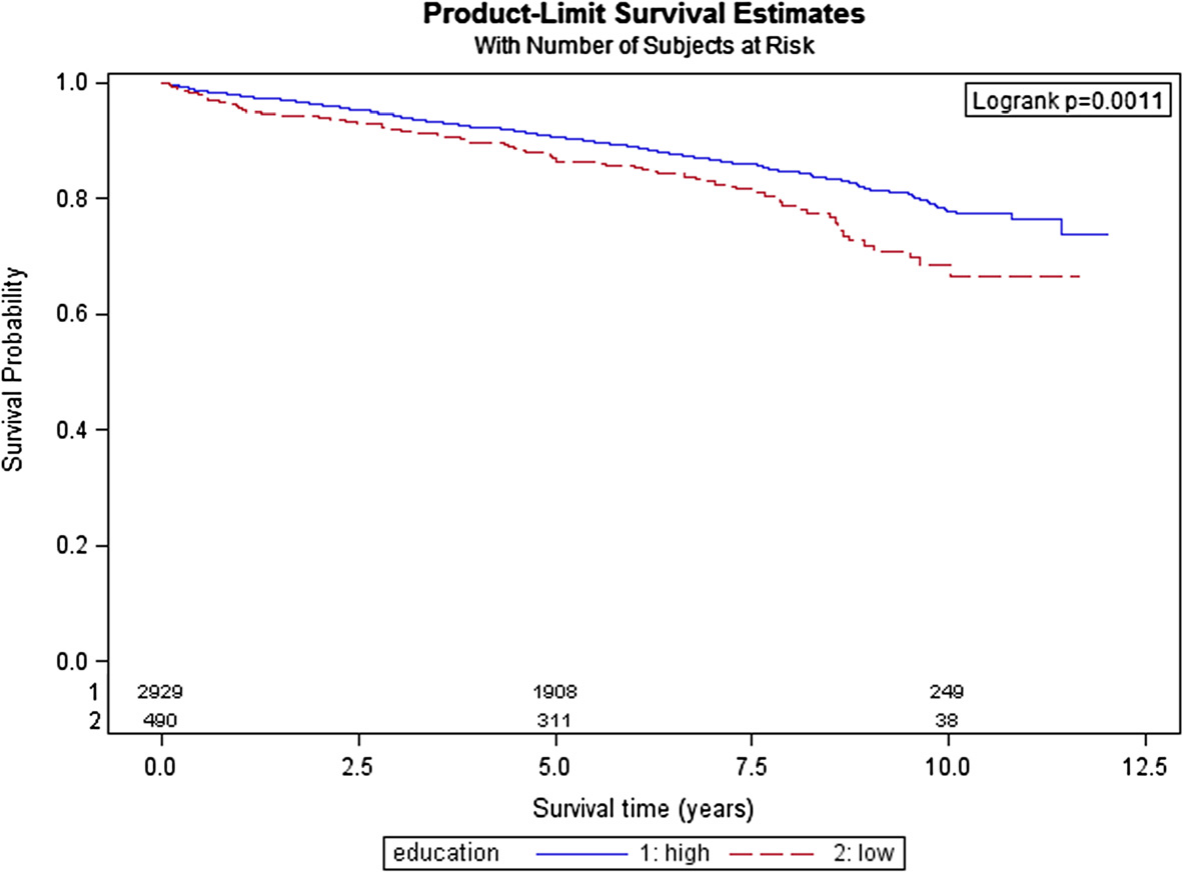
Kaplan–Meier curve and log-rank test p-value of 12-year survival for patients with low versus high education.

**Figure 2:**
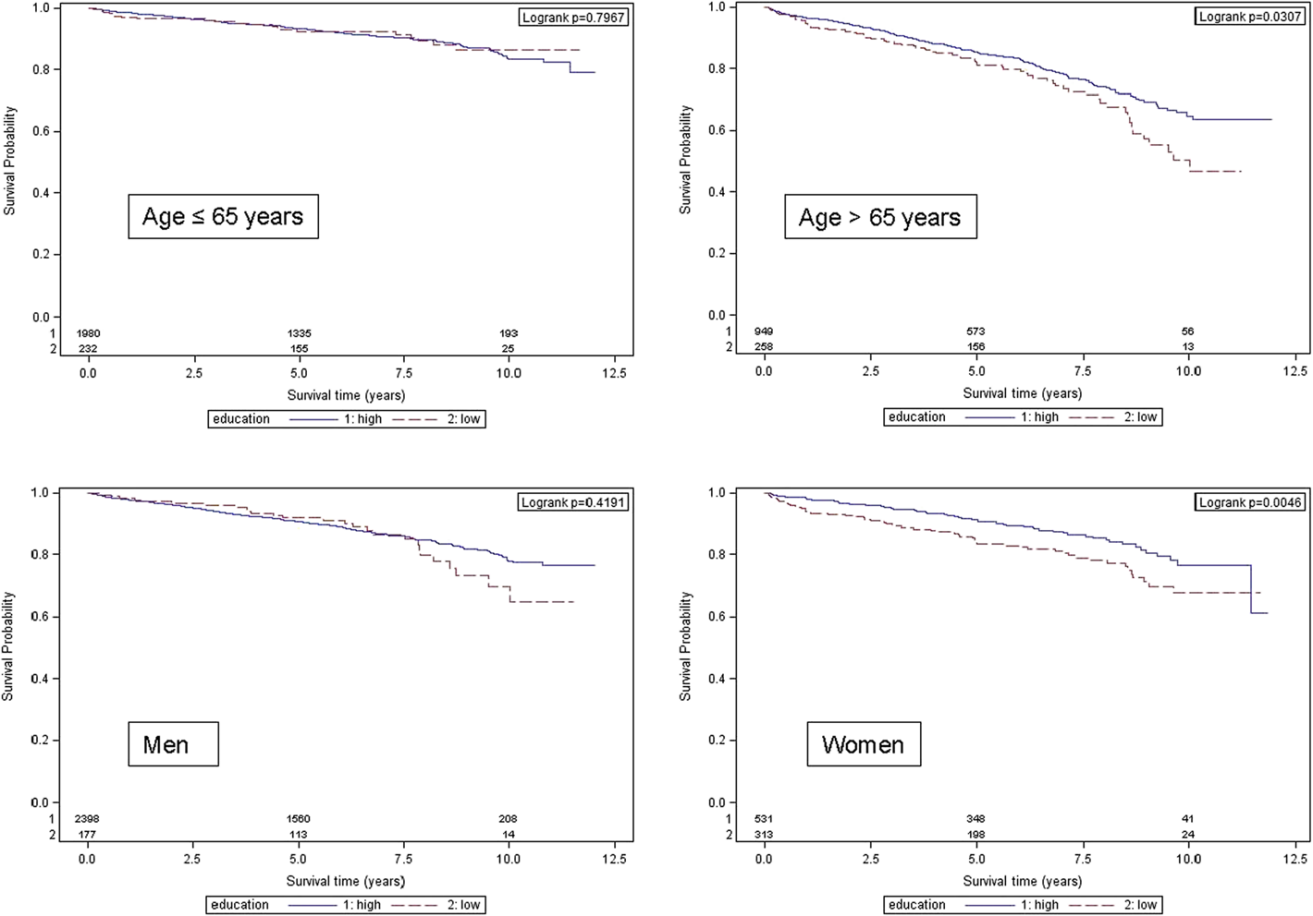
Kaplan–Meier curves and log-rank test p-values of 12-year survival for patients with low versus high education stratified by age and sex.

In the total sample, long-term mortality was 13.8% (n = 471). In the group ‘low education’, 18.6% (n = 91) died compared with 13.0% (n = 380) in the group ‘higher education’ (see Table 2). Kaplan–Meier survival curves demonstrated a significant survival difference between educational groups in the total study sample (see Figure 1). In addition, a significant survival benefit for those with higher education was found in the subgroup of patients aged > 65 years (p = 0.031) and in women (p = 0.005) (see Figure 2).

**Table 2:** Number (%) of deaths in different sex and age groups stratified by education (low versus high)

	**1-year mortality**			**5-year mortality**			**12-year mortality**		
	**Total**	**Low education**	**High education**	**Total**	**Low education**	**High education**	**Total**	**Low education**	**High education**
**Total sample** (n = 3,419)	86 (2.5)	21 (4.3)	65 (2.2)	307 (9.0)	59 (12.0)	248 (8.5)	471 (13.8)	91 (18.6)	380 (13.0)
**Age ≤ 65 years** (n = 2,212)	38 (1.7)	7 (3.0)	31 (1.6)	138 (6.2)	16 (6.9)	122 (6.2)	207 (9.4)	21 (9.1)	186 (9.4)
**Age > 65 years** (n = 1,207)	48 (4.0)	14 (5.4)	36 (3.6)	169 (14.0)	43 (16.7)	126 (13.3)	264 (21.9)	70 (27.1)	194 (20.4)
**Men**									
Total (n = 2,575)	58 (2.3)	3 (1.7)	55 (2.3)	219 (8.5)	13 (7.3)	206 (8.6)	339 (13.2)	27 (15.3)	312 (13.0)
Age ≤ 65 years (n = 1,782)	30 (1.7)	3 (2.6)	27 (1.6)	107 (6.0)	5 (4.4)	102 (6.1)	164 (9.2)	8 (7.0)	156 (9.4)
Age > 65 years (n = 793)	28 (3.5)	0	28 (3.8)	112 (14.1)	8 (12.9)	104 (14.2)	175 (22.1)	19 (30.7)	156 (21.3)
**Women**									
Total (n = 844)	28 (3.3)	18 (5.8)	10 (1.9)	88 (10.4)	46 (14.7)	42 (7.9)	132 (15.6)	64 (20.5)	68 (12.8)
Age ≤ 65 years (n = 430)	8 (1.9)	4 (3.4)	4 (1.3)	31 (7.2)	11 (9.4)	20 (6.4)	43 (10.0)	13 (11.1)	30 (9.6)
Age > 65 years (n = 414)	20 (4.8)	14 (7.1)	6 (2.8)	57 (13.8)	35 (17.9)	22 (10.1)	89 (21.5)	51 (26.0)	38 (17.4)

In addition, the following variables showed a significant (p < 0.2) association with survival: living alone, stroke, diabetes, hypertension, hyperlipidaemia, angina pectoris, any reperfusion therapy, AMI type, LVEF and in-hospital complications. Correlations among these covariables did not exceed 0.15 (Phi or Cramer V coefficient). Thus, all of them were included as covariables in the full Cox regression models.

Unadjusted Cox regression analysis resulted in a significant 1.46-fold risk of dying for patients with low education; however, the association was considerably attenuated after adjusting for sex and age (see Table 3). The final model showed a non-significant 1.16-fold increased hazard (95% CI 0.90–1.50; p = 0.256). Stratification by age revealed no significant association between educational status and mortality among patients aged 65 years or younger. In the older age group, however, there was a pronounced and significant association in favour of higher education; it could be seen in the unadjusted analysis and it remained significant even after adjustment for all significant covariates (HR 1.44; 95% CI 1.05–1.98; p = 0.023). Stratification by sex showed that the increased mortality risk for those with poor education is higher overall for women than for men (see Table 3). Women with low educational level had a significant 1.57-fold hazard of dying compared with women with higher education.

**Table 3:** **Hazard ratios [95**% **confidence interval] for mortality associated with low educational level**

	**Crude**		**Adjusted for age and sex**		**Full model **^**a**^		**Parsimonious model**	
	**HR [95% CI]**	**p-value**	**HR [95% CI]**	**p-value**	**HR [95% CI]**	**p-value**	**HR [95% CI]**	**p-value**
**Total sample** (n = 3,419)	1.46 [1.16–1.83]	0.001	1.17 [0.90–1.51]	0.236	1.16 [0.90–1.50]	0.263	1.16 [0.90–1.50]^b^	0.256
**Age ≤ 65 years** (n = 2,212)	0.94 [0.60–1.48]	0.797	0.91 [0.57–1.46]	0.701	0.88 [0.55–1.40]	0.584	0.86 [0.54–1.37]^c^	0.526
**Age > 65 years** (n = 1,207)	1.35 [1.03–1.78]	0.031	1.46 [1.07–1.99]	0.017	1.44 [1.05–1.98]	0.024	1.44 [1.05–1.98]^d^	0.023
**Men**								
Total (n = 2,575)	1.18 [0.79–1.74]	0.420	1.06 [0.72–1.56]	0.763	1.07 [0.72–1.56]	0.728	1.08 [0.73–1.61]^e^	0.701
Age ≤ 65 years (n = 1,782)	0.77 [0.38–1.57]	0.469	–	–	0.71 [0.34–1.45]	0.342	0.70 [0.34–1.42]^f^	0.319
Age > 65 years (n = 793)	1.33 [0.83–2.14]	0.242	–	–	1.44 [0.89–2.33]	0.142	1.43 [0.89–2.31]^g^	0.142
**Women**								
Total (n = 844)	1.63 [1.16–2.29]	0.005	1.25 [0.88–1.77]	0.217	1.23 [0.86–1.75]	0.252	1.24 [0.87–1.77]^h^	0.233
Age ≤ 65 years (n = 430)	1.09 [0.57–2.09]	0.796	–	–	1.10 [0.57–2.14]	0.781	1.08 [0.56–2.07]^i^	0.824
Age > 65 years (n = 414)	1.56 [1.03–2.38]	0.038	–	–	1.50 [0.97–2.32]	0.067	1.57 [1.02–2.41]^j^	0.039

^a^Adjusted for age, sex, living alone, diabetes, angina pectoris, hypertension, hyperlipidaemia, stroke, any reperfusion therapy, AMI type (STEMI, NSTEMI, bundle branch block), left ventricular ejection fraction (< 30 versus ≥ 30), any in-hospital complications.

^b^Adjusted for age, sex, diabetes, stroke, angina pectoris, hyperlipidaemia, any reperfusion therapy, AMI type, left ventricular ejection fraction.

^c^Adjusted for sex, diabetes, angina pectoris, hypertension, stroke, left ventricular ejection fraction, any reperfusion therapy, any in-hospital complications.

^d^Adjusted for sex, diabetes, hyperlipidaemia, any reperfusion therapy, AMI type, left ventricular ejection fraction.

^e^Adjusted for age, diabetes, angina pectoris, hypertension, hyperlipidaemia, stroke, any reperfusion therapy, AMI type, left ventricular ejection fraction.

^f^Adjusted for angina pectoris, hypertension, hyperlipidaemia, stroke, any reperfusion therapy, AMI type.

^g^Adjusted for angina pectoris, hyperlipidaemia, any reperfusion therapy, AMI type.

^h^Adjusted for age, diabetes, any reperfusion therapy, any in-hospital complications.

^i^Adjusted for diabetes, stroke.

^j^Adjusted for diabetes, any reperfusion therapy, any in-hospital complications.

Age-stratified analyses were also performed for different observation periods, in 1-year intervals. In those aged 65 years or older, in the fully adjusted model, HRs of approximately 1.4 were found irrespective of the observation period, reaching statistical significance only for a follow-up period of at least 9 years. In contrast, among younger people, a HR of 1.80 (95% CI 0.75–4.30; p = 0.187) was calculated for the first year after AMI, which attenuated to 1.16 in the second year and remained at around 0.90 for the rest of the observation period.

## Discussion

In this population-based study, we did not detect an overall significant negative effect of low educational level on long-term mortality after adjustment for relevant covariates. However, our study suggests that patients older than 65 years with poor education have a 46% increased risk of dying compared with more highly educated people from the same age group.

Our results can mainly be compared with previous studies that have also assessed SES at the individual level, i.e. by educational level [2,4-6,16,18,19,26,30], occupational status [8,17,18,26,28,30] and/or income [4-6,9-12,16,18,20,28]. Many of these studies have a follow-up period of 28 days, although several studies [8,9,11,17,18,28] and our results indicate that the mortality risk associated with low SES among patients up to 65 years of age decreases after the first year of follow-up.

We were able to identify 11 studies with individual SES covering a follow-up period of more than 28 days [2,4-6,10,12,16,19,20,26],[30]. Of these studies, 10 reported significantly higher long-term mortality risks for those with low SES for the whole age group under study [2,4-6,10,12,16,17,26,30], whereas our analyses indicate that this increased risk is restricted to the age group above 65 years. Some of these studies were limited to the age group younger than 66 years; however, they also detected significant associations between low SES and reduced survival [5,16,30,32].

In line with our observations, in the study by Alter et al. [20], the income–mortality gradient was attenuated by 40% after adjustment for age, and further adjustment for past cardiovascular events and current vascular risk factors resulted in a non-significant association between income and mortality. Similarly, Picciotto et al. [19] found no significant association between educational level and first-year mortality after adjustment for age and co-morbidities. However, these studies did not analyse younger and older people separately. In contrast, Rasmussen et al. [6] applied similar stratification into age groups as in our study and adjusted their Cox regression models for income. They reported that older AMI patients with high or low education did not differ regarding mortality, whereas among younger patients, those with low education had a 1.33-fold increased risk of dying. Interestingly, the Cox model not adjusted for income yielded a significant negative effect of low education on mortality in the patients aged above 65 years (HR 1.22, 95% CI 1.08–1.38).

Our finding, that among those older than 65 years, specifically women with poor education showed reduced long-term survival after AMI, is contrary to available studies which provided a stratified analysis of SES effects on mortality by sex. They showed an inverse relation between SES and 28-day case fatality only in men [11,24], in men younger than 75 years, but not in women [27], or found significant associations of SES with 5-year survival only in men [24]. However, the different findings may be influenced by the lack of comparability regarding study design and methods.

There are two main factors that are supposed to contribute to educational inequalities in mortality from AMI: inequality regarding risk factors and disparities in acute and long-term health care [4,10,16]. In our study, we have adjusted the statistical analyses for a number of risk factors, co-morbidities and acute treatment procedures, which predominantly were not evenly distributed among those with low or high education. However, we could not demonstrate that adjustment for these factors strongly attenuates the association between education and mortality.

It was not possible in our study to consider events after hospital discharge, which may account for the detected disparities in survival. Available literature indicates that patients with low SES are less likely to undergo secondary prevention measures, to attend cardiac rehabilitation and to adhere to lifestyle recommendations and medication therapy [36,37]. Adjustment to life after AMI may be more difficult for elderly people and specifically for elderly women with low education. Ho et al. [37] showed that those with a poor education were more likely to discontinue medication after AMI, and the effect of increasing age on medication therapy discontinuation was greater for females than for males. In addition, several studies have already revealed sex-related differences regarding the impact of social support and close social relationships on coping with an AMI event. For instance, it could be shown that women generally tend to use their social network less effectively than men [38] and that low education and being married significantly predict dietary non-compliance in women with hypercholesterolaemia [39]. Thus, further studies are needed to address secondary prevention measures in elderly men and women with low education in order to improve post-AMI care for these specific risk groups.

Compared with previous studies, our study has a number of strengths. First, our analyses were adjusted for factors that influence mortality after AMI and that are often not equally distributed among people with different SES. In contrast, previous studies have often not considered smoking [6,10,16,19,26], hypertension [16,26], obesity [16,19,26] or reperfusion treatment [6,10,26]. In addition, on account of the long observation period in our study, the results add new information on the stability of the effects of SES on mortality over time, which has not been investigated before. Moreover, it is one of the few studies in this field of research with a population-based sample, which allows a better generalization of the results [4,5,10,14-16]. Last but not least, this is the first study to examine the association of SES with long-term mortality after AMI in Germany.

Some limitations of the study need to be considered. The study does not include patients older than 74 years. We were not able to analyse short-term mortality as education and a number of covariates were assessed by individual interviews, which could not be performed with patients who died early after hospital admission. Also, patients who could not be interviewed for some other reason (e.g. poor general health) had to be excluded.

Further, we were not able to consider some other relevant determinants of post-AMI survival, such as the presence of a malignant disease, renal function, location and number of affected vessels and serum uric acid. Potentially relevant determinants that occurred after the index event (e.g. additional co-morbidity, compliance with secondary prevention measures, changes in vocational training and SES) could not be considered, with the exception of ‘living alone’, which was evaluated as a time-varying covariate. Thus, residual confounding cannot be excluded. Furthermore, the assessment of SES was based solely on education. Therefore, we were not able to consider any interaction with income which might be relevant [5,6]. As Molshatzki et al. [30] have illustrated, the inclusion of any single measure of SES improves long-term mortality risk prediction; however, future studies might benefit from applying multiple SES measures.

## Conclusions

Our study has demonstrated that, among German adults who have had an AMI, no overall significant negative effect of low education on long-term mortality existed after adjustment for relevant covariates. However, patients with AMI aged older than 65 years with poor education had a 44% increased risk of dying compared with more highly educated people from the same age group. These effects were stable within the 12-year observation period. In addition, furthermore, this effect was more pronounced among women older than 65 years of low education who had a 57% increased risk of dying compared to those with high education.

More research on the association between education and long-term mortality after first-time AMI is clearly needed. It is important to understand in more detail why long-term mortality is especially high in elderly patients with low education. It can by hypothesized that appropriate risk factor management, which could decrease the risk of mortality, may be more difficult for elderly people with low education. Also, during the long follow-up period, additional co-morbidities may have come up specifically among patients with low education, making it necessary to provide them with more health care according to their increased needs. It may be useful, for example, to intensify the health care of older patients with poor education, e.g. to provide nurse-based case management after hospital discharge in order to facilitate secondary prevention measures [40].

## Competing interests

The authors declare that they have no competing interests.

## Authors’ contributions

IK, CM, AP and AM conceived the study. IK performed the statistical analysis and drafted the manuscript. PQ contributed to data analysis. BK, WS, CM and AM contributed to the interpretation of data. HG, MH, CM, BK and WS contributed to data acquisition. AM helped in drafting the manuscript. PQ, CM, AP, MH, BK, HG and WS critically revised the manuscript. All authors read and approved the manuscript.
